# The Polyamine Putrescine Is a Positive Regulator of Group 3 Innate Lymphocyte Activation

**DOI:** 10.4049/immunohorizons.2200097

**Published:** 2023-01-13

**Authors:** Prakash Sah, Lauren A. Zenewicz

**Affiliations:** Department of Microbiology and Immunology, College of Medicine, University of Oklahoma Health Sciences Center, Oklahoma City, OK; Department of Microbiology and Immunology, College of Medicine, University of Oklahoma Health Sciences Center, Oklahoma City, OK

## Abstract

Group 3 innate lymphocytes (ILC3s) rapidly respond to invading pathogens or inflammatory signals, which requires shifting cellular metabolic demands. Metabolic adaptations regulating ILC3 function are not completely understood. Polyamines are polycationic metabolites that have diverse roles in cellular functions and in immunity regulate immune cell biology, including Th17 cells. Whether polyamines play a role in ILC3 activation is unknown. In this article, we report that the polyamine synthesis pathway is important for ILC3 activation. IL-23–activated mouse ILC3s upregulate ornithine decarboxylase, the enzyme catalyzing the rate-limiting step of the conversion of ornithine to putrescine in polyamine synthesis, with a subsequent increase in putrescine levels. Inhibition of ornithine decarboxylase via a specific inhibitor, α-difluoromethylornithine, reduced levels of IL-22 produced by steady-state or IL-23–activated ILC3s in a putrescine-dependent manner. Thus, the polyamine putrescine is a positive regulator of ILC3 activation. Our results suggest that polyamines represent a potential target for therapeutic modulation of ILC3 activation during infection or inflammatory disorders.

## Introduction

Innate lymphocytes (ILCs) are a heterogenous group of non–B, non–T lymphocytes enriched at mucosal surfaces and are critical regulators of tissue homeostasis, inflammation, and resistance to pathogens (1, 2). Group 3 innate lymphocytes (ILC3s) are innate counterparts to Th17 cells due to shared lineage-defining transcription factors and secreted cytokines. ILC3s are activated by innate cytokines, most notably IL-23 or IL-1β, and on activation rapidly secrete high levels of effector molecules, including their most critical cytokine, IL-22 (3). ILC3s are particularly enriched in the gastrointestinal (GI) tract, where they regulate barrier resistance, mainly through IL-22–mediated antimicrobial and tissue-protective responses (3, 4).

Metabolism of immune cells is integral to their function. On activation, immune cells must rapidly alter their metabolism to meet the energy demands for growth, proliferation, and function (5, 6). Although cellular metabolism of T cells has been well studied, much less is known about ILC3s. Recent work by others has found that core metabolic pathways, such as glycolysis, are essential for proper ILC3 activation (7). Several peripheral metabolic pathways, which are linked to core metabolism, are also important for immune cell function (8). The polyamine synthesis pathway generates small cationic metabolites, including putrescine, spermidine, and spermine from the amino acid arginine (8, 9). Polyamines regulate diverse cellular functions, including proliferation, gene regulation, protein synthesis, and ion transport (9). Polyamines are also important for immune cells, such as regulation of NK cell metabolism and function, macrophage polarization, Th cell proliferation and differentiation, and Th17 cell function (10–15). The mechanisms ascribed to polyamine function in immune cells include regulation of transcriptional and epigenetic changes, hypusination, and rewiring of core metabolic pathways such as glycolysis and oxidative phosphorylation (11–15). In Th17 cells, polyamines regulate transcriptomic and epigenetic rewiring, and inhibition of polyamine synthesis leads to decreased Th17-associated cytokine production (14). Given the shared biology between Th17 cells and ILC3s, we hypothesized that polyamines regulate ILC3 activation.

In this article, we report that polyamines are important for ILC3 activation. Activation of mouse ILC3s by IL-23 upregulated *Odc1*, which encodes ornithine decarboxylase (ODC), the enzyme that catalyzes the rate-limiting step in polyamine biosynthesis, the conversion of ornithine to putrescine. Activated ILC3s had increased levels of the polyamine putrescine. Inhibition of ODC using a specific inhibitor, α-difluoromethylornithine (DFMO), led to decreased IL-22 production in steady-state or IL-23–activated ILC3s, which was rescued by addition of exogenous putrescine. These results show that polyamines, especially putrescine, are positive regulators of ILC3 activation.

## Materials and Methods

### Cell line

MNK-3 cells clone B3 cells, derived from single-cell cloning of the ILC3-like cell line MNK-3 (16), were maintained in DMEM (Corning, Tewksbury, MA) with 10% heat-inactivated FBS (Gemini Bio-Products, West Sacramento, CA), 2 mM GlutaMAX (Life Technologies, Carlsbad, CA), 1 mM sodium pyruvate (GE Healthcare Hyclone, Logan, UT), 55 μM 2-ME (Sigma, St. Louis, MO), 10 mM HEPES (Corning), 50 μg/ml gentamicin (Amresco, Solon, OH), 100 U/ml penicillin (Gemini Bio-Products), 100 U/ml streptomycin (Gemini Bio-Products), and 10 ng/ml recombinant mouse IL-7 (PeproTech, Rocky Hill, NJ).

### ILC3 activation and treatment

Cells were grown in the presence of DFMO (Tocris Biosciences, Bristol, U.K.) for 3 d to deplete polyamines. For putrescine rescue experiments, putrescine (Sigma) was added together with DFMO. Untreated cells were used as a control. On day 3, cells were harvested and counted. An equal number of DFMO-treated and untreated cells were stimulated. For ELISA and intracellular cytokine staining (ICS) experiments, cells were stimulated for 18 and 5 h, respectively. Where noted, DFMO with or without putrescine was added during stimulation. Unless otherwise stated, DFMO was used at 500 μM. Putrescine was used at 500 μM. Recombinant mouse IL-23 (BioLegend, San Diego, CA) and IL-1β (BioLegend) were used at 50 and 20 ng/ml, respectively. PMA (Sigma) and ionomycin (Sigma) were used at 5 and 0.5 μg/ml, respectively.

### Gene expression analysis

Cells were stimulated with IL-23 or left untreated, and RNA was purified using a Direct-zol RNA miniprep plus kit (Zymo Research, Irvine, CA). RNA expression profiling was performed via NanoString nCounter Analysis Technology (NanoString, Seattle, WA) using an nCounter mouse metabolism pathways panel with 748 genes and 20 internal reference genes. Data were analyzed using the nSolver 4.0 software package. In brief, raw transcript counts were normalized using negative and positive synthetic sequences provided within each code set to account for background noise and technical variation, respectively. Differential gene expression between untreated and IL-23–treated cells was examined. Genes upregulated or downregulated 2-fold or more with adjusted *p* < 0.05 were identified as modulated by IL-23.

### Real-time RT-PCR

Cells were harvested in TriPure (Roche, Nutley, NJ), and RNA was prepared according to the manufacturer’s protocol. RNA was DNase treated (Roche), and cDNA was generated by reverse transcription using EasyScript Plus (Lambda Biotech, St. Louis, MO) with oligo dT as the primer. cDNA was used as the template in a real-time PCR using Integrated DNA Technologies (IDT, Coralville, IA) or ABI TaqMan primer-probes sets (Thermo Fisher, Waltham, MA) on a QuantStudio5 real-time PCR instrument (Thermo Fisher). Primer-probe sets used were *Hprt* (Mm.PT.39a.22214828; IDT), *Il22* (Mm.PT.58.44024580.g; IDT), and *Odc1* (Mm02019269_g1; Thermo Fisher). cDNA was semiquantitated using the ΔC_T_ method with *Hprt* as an internal control for all samples.

### Western blotting

Cells were washed with cold PBS and lysed with cell lysis buffer (Cell Signaling Technology, Danvers, MA). Cell lysates were separated by SDS-PAGE on a 4–15% gradient gel (Bio-Rad, Hercules, CA) followed by transfer to an Immobilon-P PVDF membrane (EMD Millipore, Billerica, MA) using a wet transfer method. The protein-transferred membrane was blocked with 5% milk in TBS with 0.1% Tween 20 and then incubated with the manufacturer’s recommended concentration of primary Ab (1:1000) overnight with rocking at 4°C. Primary Abs used were anti-ODC (ab97395; Abcam) and anti-actin (3700; Cell Signaling Technology). Blots were then washed and incubated with the appropriate species-specific HRP secondary Ab (1:2000) for 1 h. Blots were developed using Pierce ECL2 Western blotting Substrate (Thermo Fisher) and imaged using a ChemiDoc MP imaging system (Bio-Rad). For reblotting with another Ab, blots were stripped using a Restore Western blot Stripping Buffer (Thermo Fisher), then washed and reblocked and used as indicated earlier.

### Proliferation and viability assays

Cells were labeled according to the manufacturer’s protocol with 5 μM CFSE (eBioscience, San Diego, CA) and cultured with or without DFMO for 3 d. Cells were harvested on each day, stained with eFluor780 fixable viability dye (Thermo Fisher), and analyzed by flow cytometry on a Stratedigm S1200Ex flow cytometer (Stratedigm, San Jose, CA). Data were analyzed using FlowJo v.10.6 (Tree Star, Ashland, OR).

### Intracellular cytokine staining

Cells were treated with IL-23 as indicated in the presence of brefeldin A (BFA; eBioscience) for 5 h. Cells were stained with eFluor780 fixable viability dye and then were intracellularly stained with anti–IL-22 Ab (clone IL22JOP; eBioscience) according to the manufacturer’s protocol and analyzed by flow cytometry.

### ELISA

Mouse IL-22 ELISA (Antigenix America, Huntington Station, NY) was performed according to the manufacturer’s protocol.

### Polyamine analysis

Cells were washed with cold PBS and mixed with 0.1% heptafluorobutyric acid followed by sonication. Putrescine-d8 was included as an internal standard. Acetonitrile was added to precipitate proteins and centrifuged at 15,000 × *g* for 15 min at 4°C. Supernatant was analyzed by HPLC-mass spectrometry (MS) on an Ultimate 3000 HPLC and TSQ Quantis triple-quadrupole mass spectrometer (Thermo Scientific).

### Statistical analysis

Values are expressed as mean ± SD. Statistical analysis was performed with Prism 9.0.2 (San Diego, CA). For two-way comparisons, an unpaired *t* test was used. For multiple comparisons, one-way or two-way ANOVA with Tukey’s or Sidak’s multiple comparison tests was used. Significance was defined as *p ≤ 0.05, **p < 0.01, ***p < 0.001, and ****p < 0.0001. Differences that were not significant (*p* > 0.05) were marked as “ns.”

## Results

### The key enzyme in polyamine synthesis, ODC, is upregulated in IL-23–activated ILC3s

**FIGURE 1.**
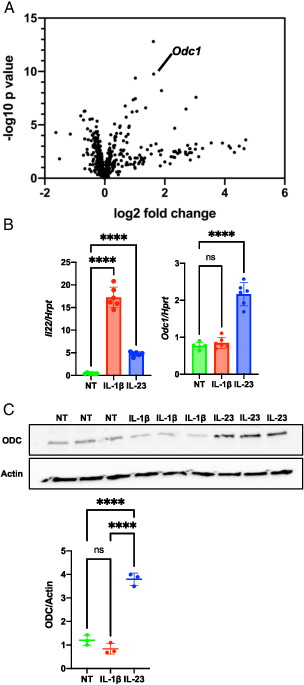
ODC is upregulated in IL-23–activated ILC3s. (**A**) MNK-3 cells were treated with IL-23 for 4 h or left untreated and analyzed by NanoString nCounter analysis using a mouse metabolism panel with 748 genes and 20 internal reference genes. Shown is a volcano plot with the *Odc1* marked. (**B**) MNK-3 cells were treated with IL-23 or IL-1β for 4 h or left untreated (NT). *Il22* and *Odc1* mRNA levels were semiquantitated by real-time RT-PCR. Each point represents one well, and bars indicate mean. *n* = 6. Data are representative of three independent experiments. (**C**) MNK-3 cells were treated with IL-23 or IL-1β for 18 h or left NT, and cell lysates were analyzed by Western blotting to examine ODC and actin levels. Each point represents one well, and lines indicate means. *n* = 3. Data are representative of two independent experiments. ****p < 0.0001. Differences that were not significant (*p* > 0.05) are marked as “ns.”

To examine whether cellular metabolism is altered in activated ILC3s, we performed a targeted gene expression analysis. Because ILC3s are very rare cells found at low frequency even in mucosal tissues, we used a mouse ILC3-like cell line, MNK-3, because these cells are highly transcriptionally similar to primary mouse ILC3s (16). These cells produce low levels of IL-22 at homeostasis, but on stimulation with IL-1β or IL-23 produce copious amounts of IL-22 (16). ILC3s were stimulated with the cytokine IL-23 for 4 h or left untreated and then subjected to NanoString analysis using a metabolism gene-focused panel with 748 genes. We found that 19 genes were upregulated and none were downregulated >2-fold compared with their levels in unstimulated cells (Table I). One gene of particular interest due to high statistical significance was *Odc1*, which encodes ODC (Fig. 1A). *Odc1* upregulation by IL-23 was confirmed by real-time RT-PCR (Fig. 1B), and ODC protein levels were significantly increased as well (Fig. 1C). Activation by another innate cytokine, IL-1β, did not increase levels of *Odc1* or ODC (Fig. 1B, 1C). Our data show that ODC is upregulated in ILC3s under select activation conditions.

**Table I.** mRNA levels modulated in MNK-3 cells by IL-23 treatment

mRNA	Log2 Fold Change	Linear Fold Change	*p* Value	Benjamini-Yekutieli *p* Value
*Il2ra*	1.62	3.08	1.62E−13	5.83E−10
*Odc1*	1.63	3.09	1.79E−10	3.22E−7
*Gzmb*	1.02	2.03	4.21E−10	5.07E−7
*Myd88*	1.89	3.71	6.11E−9	5.51E−6
*Cyp1b1*	3.04	8.23	2.66E−8	1.92E−5
*Gda*	1.02	2.03	3.45E−8	2.07E−5
*Stat5a*	1.36	2.57	2.58E−7	0.000103
*Cyp4a12a*	2.71	6.55	3.3E−7	0.000119
*Acox1*	1.07	2.1	5.34E−7	0.000148
*Ptpn5*	2.33	5.02	2.07E−5	0.00276
*Haao*	4.7	26	0.00026	0.0171
*Thbs2*	3.55	11.7	0.00049	0.0266
*Ms4a1*	3.92	15.1	0.00051	0.0273
*Pklr*	1.71	3.26	0.00061	0.0312
*Slc16a11*	3.04	8.23	0.00064	0.0325
*Cd14*	2.76	6.79	0.00084	0.0395
*Arid2*	1.54	2.91	0.00084	0.0395
*Cyp4a10*	4.14	17.6	0.00096	0.0438
*Cacna1a*	4.48	22.2	0.00099	0.0438

### IL-23–activated ILC3s have elevated levels of putrescine

**FIGURE 2.**
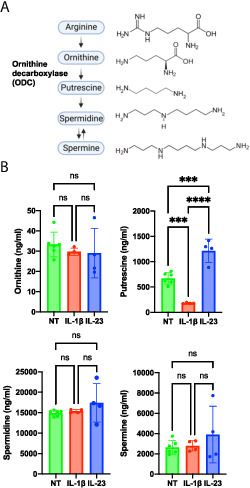
Polyamines are increased in IL-23–activated ILC3s. (**A**) Polyamine synthesis pathway. ODC catalyzes the rate-limiting step of the conversion of ornithine to putrescine in the polyamine synthesis pathway. Putrescine acts as a substrate for the synthesis of spermidine, which in turn acts a substrate for the synthesis of spermine. (**B**) MNK-3 cells were activated by IL-1β (*n* = 4), IL-23 (*n* = 4), or left untreated (NT) (*n* = 6) for 18 h. Cells were harvested and subjected to targeted metabolomics analysis by HPLC-MS. Each point represents one well, and bars indicate mean. Data presented are representative of two independent experiments. ***p < 0.001, ****p < 0.0001. Differences that were not significant (*p* > 0.05) are marked as “ns.”

ODC catalyzes the rate-limiting step in polyamine synthesis in the conversion of ornithine to putrescine (Fig. 2A) (8). Putrescine is then converted to the two other polyamines commonly found in eukaryotes, spermidine and spermine. To determine whether higher levels of ODC result in increased polyamine biosynthesis in ILC3s, we quantitated ornithine and the polyamines putrescine, spermidine, and spermine in resting, IL-1β–, and IL-23–activated ILC3s. No difference was detected in levels of the polyamine precursor ornithine between resting and activated ILC3s (Fig. 2B). Increased levels of putrescine, but not spermidine or spermine, were observed in IL-23–activated ILC3s compared with resting cells. The increase in putrescine was consistent with increased ODC levels in IL-23–activated cells. In contrast, we detected decreased putrescine levels in IL-1β–activated ILC3s compared with resting cells, although *Odc1* and ODC levels were not significantly changed. Thus, IL-23–activated ILC3s have increased levels of the polyamine putrescine.

### Polyamines are important for ILC3 activation

**FIGURE 3.**
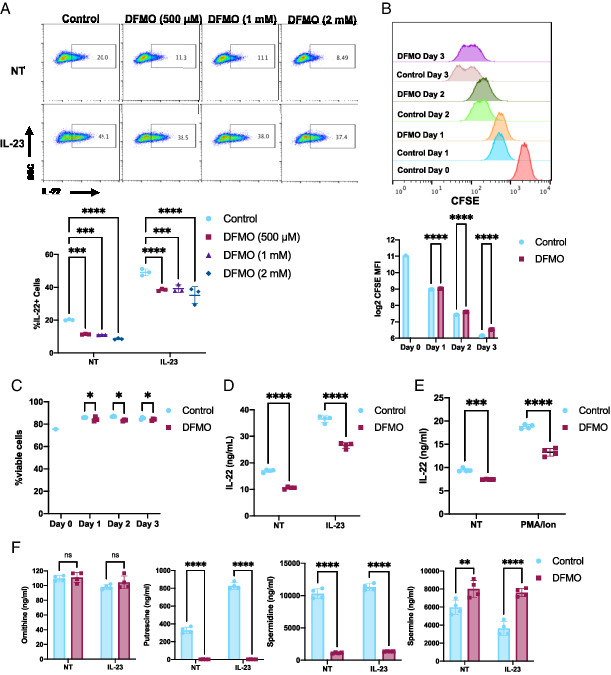
Inhibition of polyamine biosynthesis reduces ILC3 activation. (**A**) MNK-3 cells grown with the indicated concentration of DFMO were stimulated with IL-23 or not (NT) for 5 h in the presence of BFA and then analyzed by ICS and FACS. Shown are representative FACS plots and summary data. Each point represents one well, and lines indicates means. *n* = 3. Data are representative of two independent experiments. (**B** and **C**) CFSE-labeled MNK-3 cells were grown in the presence or absence of 500 μM DFMO over 3 days. Each day, cells were analyzed for CFSE and viability staining by FACS. Shown are (B) representative histogram plots and CFSE mean fluorescence intensity (MFI) and (C) viability data. Each point represents one well, and lines indicate means. *n* = 3. Data are representative of three independent experiments. (**D** and **E**) MNK-3 cells grown with or without 500 μM DFMO were stimulated with IL-23 or 5 μg/ml PMA and 0.5 μg/ml ionomycin or NT for 18 h. IL-22 was quantitated in the supernatants by ELISA. Each point represents one well, and lines indicate means. *n* = 4. Data are representative of three independent experiments. (**F**) MNK-3 cells grown with or without 500 μM DFMO were stimulated with IL-23 or NT for 18 h, and cells were subjected to metabolomics analysis by HPLC-MS. Each point represents one well, and bars indicate means. *n* = 4. *p ≤ 0.05, **p < 0.01, ***p < 0.001, ****p < 0.0001. Differences that were not significant (*p* > 0.05) are marked as “ns.”

To test whether polyamine biosynthesis is important for ILC3 activation, we treated the ILC3s with different concentrations (500 μM, 1 mM, 2 mM) of the ODC inhibitor, DFMO, for 3 d to deplete polyamines, similar to others’ T cell studies (10, 14). MNK-3 cells produce low levels of IL-22–like ILC3s found in vivo in immune homeostasis, and DFMO reduced these resting IL-22 levels (Fig. 3A). Cells were then stimulated with IL-23, and ILC3 activation was assessed by quantitating IL-22 production, one of the most highly upregulated and critical effector molecules produced by activated ILC3s (3). IL-23 stimulation increased the percentage of cells producing IL-22, and this increase was significantly reduced in DFMO-treated cells (Fig. 3A). At these concentrations, the effect of DFMO was similar, and therefore we performed all subsequent studies at the lowest concentration of 500 μM.

Although DFMO treatment resulted in a small decrease in cell proliferation and viability, these changes are likely not biologically significant (Fig. 3B, 3C). Nevertheless, to minimize any effect of DFMO on proliferation or viability, we used an equal number of DFMO-treated or untreated cells for activation experiments. We next measured secreted IL-22 in DFMO-treated ILC3s after IL-23–mediated activation. DFMO treatment reduced IL-22 secretion by ILC3s in both resting and IL-23–stimulated cells (Fig. 3D). To test whether DFMO treatment affected ILC3 activation that bypasses cytokine signaling, we activated DFMO-treated or untreated cells by PMA and ionomycin. DFMO treatment reduced PMA and ionomycin-induced IL-22 levels (Fig. 3E). To determine whether DFMO treatment reduced polyamine levels, we quantitated ornithine and the polyamines putrescine, spermidine, and spermine in DFMO-treated resting and IL-23–activated ILC3s. DFMO treatment led to reduced putrescine and spermidine levels (Fig. 3F). However, spermine levels increased in DFMO-treated ILC3s. Thus, our data show inhibition of polyamine synthesis leads to reduced ILC3 activation.

### Putrescine rescues DFMO-mediated reduction in IL-22 production by ILC3s

**FIGURE 4.**
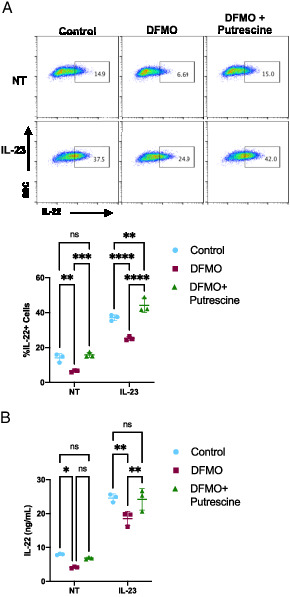
Putrescine rescues DFMO-mediated reduction of ILC3 activation. (**A**) MNK-3 cells grown in the presence or absence of 500 μM DFMO with or without 500 μM putrescine were stimulated or not (NT) with IL-23 for 5 h in the presence of BFA followed by ICS and FACS analysis. Shown are representative FACS plots and summary data. Each point represents a well, and lines indicate means. *n* = 3. Data are representative of three independent experiments. (**B**) MNK-3 cells were grown in the presence or absence of 500 μM DFMO with or without 500 μM putrescine. Cells were stimulated with IL-23 or left NT for 18 h, and IL-22 in the supernatants was quantitated by ELISA. Each point represents one well, and lines indicate means. *n* = 3. Data are representative of three independent experiments. *p ≤ 0.05, **p < 0.01, ***p < 0.001, ****p < 0.0001. Differences that were not significant (*p* > 0.05) are marked as “ns.”

Putrescine is the product of the ODC-catalyzed reaction in the polyamine synthesis pathway and was reduced in ILC3s by DFMO treatment (Fig. 3F). To show that the effect of DFMO is specific to the polyamine synthesis pathway, we added exogenous putrescine during DFMO treatment of cells and then activated cells with IL-23 and examined IL-22 levels. The addition of exogenous putrescine rescued the percentage of IL-22–producing cells in both resting and IL-23–activated DFMO-treated ILC3s to levels similar to control cells (Fig. 4A). Similarly, we also assessed secreted IL-22 and found that exogenous putrescine rescued secreted IL-22 levels in both steady-state and IL-23–activated DFMO-treated ILC3s (Fig. 4B). These data suggest that the polyamine pathway is an important positive regulator of ILC3 activation.

## Discussion

Immune cells undergo metabolic reprograming to support their activation in response to invading pathogens or inflammatory signals (5, 6). This involves core metabolic pathways that generate energy and associated peripheral pathways (8). In this study, we investigated the role of a peripheral metabolic pathway, the polyamine synthesis pathway, in activation of ILC3s and found that the polyamine putrescine is a positive regulator of ILC3 activation.

The rate-limiting step in the polyamine synthesis pathway, conversion of ornithine to putrescine, is catalyzed by ODC and thus controls de novo polyamine synthesis (8). We observed increased *Odc1* and ODC levels in IL-23–activated ILC3s. Importantly, *Odc1* is reported to be selectively expressed in primary mouse ILC3s in two independent studies comparing ILC transcriptional profiles (17, 18), and these data suggest that *Odc1* may have potentially distinct roles in different ILC subsets. Activation by another innate cytokine, IL-1β, did not increase levels of *Odc1*, ODC, or polyamines, suggesting that the increase in ODC levels is not a general response to ILC3 activation but regulated by a distinct signaling pathway. Aryl hydrocarbon receptor (AHR) can act as a direct transcriptional activator of *Odc1* in nonimmune cells (19). Given the central role of AHR in regulating ILC3 activation, AHR could potentially regulate *Odc1* expression during ILC3 activation. Consistent with increased levels of ODC, IL-23–activated cells also showed increased levels of putrescine. The lack of an observable change in spermidine or spermine levels may be because of diversion of putrescine to alternative fates, other than conversion to spermidine (14, 20).

DFMO is an ornithine analogue that binds to ODC irreversibly in its catalytic site, making it a highly specific inhibitor of ODC (21). DFMO treatment reduced the levels of the polyamines putrescine and spermidine in ILC3s, whereas spermine levels increased. DFMO is known to reduce putrescine and spermidine levels with little effect on spermine levels (21). DFMO treatment reduced IL-22 production of steady-state and IL-23–activated ILC3s, which was rescued by adding putrescine, suggesting this polyamine is an important positive regulator of ILC3 activation.

Polyamines are polycationic molecules that have diverse roles in cellular metabolism, including gene expression, RNA structure, protein synthesis, autophagy, and proliferation (9). Recent studies have described roles for polyamines in immune cells, such as NK cells, macrophages, dendritic cells, and T cells, including Th17 cells (9, 11, 13–15). In Th17 cells, the mechanisms of polyamine function are through mediating transcriptional and epigenetic changes (14). In activated NK cells, de novo polyamine synthesis supports the core metabolic pathways, glycolysis and oxidative phosphorylation (15). A key function of ILC3s is to rapidly produce cytokines. In ILC3s, polyamines may increase cytokine gene accessibility or have downstream effects on cytokine mRNA stability. Alternatively, or additionally, polyamines could rewire core metabolic pathways such as glycolysis and/or oxidative phosphorylation that are important for ILC3 activation (7). Future studies will investigate the mechanism(s) of polyamine-dependent regulation of ILC3 activation.

Targeting cellular metabolism of immune cells by small-molecule inhibitors could provide new ways of treating infectious diseases and inflammatory disorders (6). ILC3s are important regulators of tissue homeostasis and host resistance to bacterial infections. ILC3s are often dysregulated in inflammatory diseases such as inflammatory bowel disease or psoriasis (3, 22). Colonic ILC3s from ulcerative colitis and Crohn’s disease patients produce higher IL-22 levels than healthy control subjects, and this excessive IL-22 can cause GI inflammation (23, 24). In psoriasis patients compared with healthy individuals, ILC3s are increased in skin lesions and blood and represent an important source of IL-22 that contributes to the pathogenesis of psoriasis (22, 25). Our identification of the polyamine pathway as an important positive regulator of ILC3 activation leads us to propose that polyamine metabolism could be targeted to control ILC3 activation in such inflammatory diseases. DFMO is a well-tolerated and Food and Drug Administration–approved drug for treating trypanosome infection and female facial hirsutism (21). In contrast, supplementation with polyamines, especially putrescine, may augment the host innate immune response against bacterial pathogens, although careful examination is warranted because polyamines can also impact bacterial growth and metabolism (26). Spermidine has recently been shown to have a protective role in colitis through interactions with epithelial cells (27). Thorough assessment of the effects of polyamine synthesis inhibition or polyamine supplementation in the GI tract should include examination of the immune cells, epithelial cells, and commensals.

A limitation to our study is that we have not examined primary ILC3s. A new complementary study has shown a role for polyamines in primary mouse ILC3s (28), in addition to also examining the MNK-3 cell line we have used in our study. They observed that ILC3s isolated from the small intestine when stimulated with IL-23 produced higher levels of IL-22 when 10 mM putrescine was also added. In addition, using a cre-flox system to delete *Odc1* in *Rorc*-expressing cells, they found that ODC had a role in in vivo IL-22 production by ILC3s during two distinct models of colitis. However, it still needs to be determined whether inhibition of polyamine synthesis through DFMO alters in vivo ILC3 activation, which has greater potential for translation to patients than results from genetically altered mice.

In this study, we have shown that the polyamine putrescine is a positive regulator of ILC3 activation. Putrescine levels are increased in activated ILC3s and when polyamine synthesis is inhibited, homeostatic and activated ILC3s produce less cytokine. Elucidation of metabolic adaptations during ILC3 activation is important for understanding ILC3 function and providing a rationale for new or co-opting existing therapeutics to combat GI inflammation.
